# Interference With Redox Homeostasis Through a G6PD-Targeting Self-Assembled Hydrogel for the Enhancement of Sonodynamic Therapy in Breast Cancer

**DOI:** 10.3389/fchem.2022.908892

**Published:** 2022-05-04

**Authors:** Cuiqing Huang, Yuan Xu, Duo Wang, Zerong Chen, Weimin Fang, Changzheng Shi, Zeyu Xiao, Liangping Luo

**Affiliations:** ^1^ The Guangzhou Key Laboratory of Molecular and Functional Imaging for Clinical Translation, The First Affiliated Hospital of Jinan University, Guangzhou, China; ^2^ Department of Ultrasound, Guangdong Women and Children Hospital, Guangzhou, China; ^3^ The Medical Imaging Center, The First Affiliated Hospital of Jinan University, Guangzhou, China

**Keywords:** self-assembled hydrogel, sonodynamic therapy, glucose-6-phosphate dehydrogenase, RRx-001, redox homeostasis

## Abstract

Sonodynamics has emerged as a new potential therapy for breast cancer in recent years. However, GSH-mediated redox systems in cancer cells make them tolerable to oxidative stress-related therapy. Herein, in this study, with G6PD, the gatekeeper enzyme of the pentose phosphate pathway, as the regulative target, a self-assembled thermosensitive chitosan-pluronic hydrogel coloaded with ICG (sono-sensitive agent) and RRx-001 (IR@CPGel) was successfully prepared to enhance SDT through interference with redox homeostasis. Both *in vitro* and *in vivo* antitumor investigations verified that when integrated with sonodynamic therapy applied in breast cancer treatment, local administration of IR@CPgel could enhance ROS generation under LIFU irradiation and trigger the intrinsic apoptotic pathway of cancer cells, thus effectively inhibiting tumor growth in a safe manner. Moreover, RRx-001 may interfere with redox homeostasis in cancer cells by downregulating G6PD expression. Due to this redox imbalance, proapoptotic signals, such as P21 and P53, were enhanced, and metastasis-related signals, including MMP-2, ZEB1 and HIF-1α, were effectively reduced. Taken together, this work aimed to enhance the efficacy of sonodynamic therapy through local administration of self-assembled IR@CPGel to interfere with redox homeostasis and thus amplify the oxidative stress microenvironment in tumor tissues. In a word, this work provides a new strategy for the SDT enhancement in breast cancer therapy.

## Introduction

Breast cancer, with a high incidence among women, is the leading cause of mortality among women throughout the world (Carioli et al., 2017; Azamjah et al., 2019; Wang et al., 2019). Present clinical therapy is still focused on resection surgery and chemotherapy, which are bothered by a series of issues, such as low selectivity, severe side effects or toxicity, and poor prognosis. In recent years, sonodynamic therapy (SDT) has emerged as an alternative strategy for the noninvasive treatment of solid tumors, including breast cancer (Xing et al., 2021; Zhang et al., 2021). SDT is a therapy modality similar to photodynamic therapy (PDT), in which light is replaced by ultrasound to activate the sensitizer (He et al., 2019; Hu et al., 2020). Therefore, the penetration depth of SDT (several tens of centimeters) is superior to that of PDT, which enables the wide application of SDT in various deeper-seated tumors (Wang et al., 2020). The anticancer efficacy of SDT is realized specifically through the oxidative stress induced by the overgeneration of reactive oxygen species (ROS) under simultaneous irradiation with low-intensity focused ultrasound (LIFU) combined with a sono-sensitizer. However, due to the hypoxic microenvironment and resistance mechanisms in tumor tissues, the therapeutic efficiency of SDT is partially confined (Kuroki et al., 2007; Costley et al., 2015).

Glutathione (GSH) is a critical element of cellular redox homeostasis. By enhancing GSH levels, cancer cells may scavenge excessive reactive oxygen species (ROS) and detoxify xenobiotics (He et al., 2017; Niu et al., 2021). GSH is thus emerging as a new potential target for enhancing SDT (Tang et al., 2015). Indeed, related studies have proven that the decrease in GSH levels in cancer cells makes them more susceptible to oxidative stress (Traverso et al., 2013; Matés et al., 2020). In addition, GSH depletion has also been demonstrated to enhance the antitumor efficacy of treatment strategies based on oxidative stress, such as PDT, SDT, chemodynamic therapy, and ferroptosis (Lin et al., 2018; Sun et al., 2018; Fu et al., 2019; Hu et al., 2019). Glucose-6-phosphate dehydrogenase (G6PD) is a key gatekeeper enzyme of the pentose phosphate pathway for the maintenance of redox homeostasis mediated by GSH-based systems that require NADPH (Catanzaro et al., 2015; Cho et al., 2018). To date, Okamoto et al. verified that the upregulation of G6PD and other NADPH-related redox genes could be detected in chemoresistant spheroids, indicating that G6PD may be a potential target for interfering with redox homeostasis in cancer cells to fulfill the enhancement of SDT (Yamawaki et al., 2021).

RRx-001 is a small molecular drug with pan-epigenetic properties that may induce oxidative stress by triggering ROS generation in hypoxic cancer cells (Zhao et al., 2015; Das et al., 2016). Preclinical investigations on different animal models and patients have confirmed its antitumor efficacy (Reid et al., 2015; Kim et al., 2016; Oronsky et al., 2017). Apart from epigenetic alterations, RRx-001 also exhibits pleiotropic physiological functions in redox homeostasis, apoptosis, etc., in cells through regulation of different signaling pathways, such as G6PD, Nrf2, P53, PARP cleavage, and HIF-1α (Ning et al., 2015; Oronsky et al., 2016). Therefore, the codelivery of G6PD inhibitors (such as RRx-001) and sonosensitizers in tumor tissues to deplete GSH and disrupt redox homeostasis may maximally enhance the treatment efficiency of SDT in breast cancer.

Local administration of self-assembled hydrogels for drug delivery has made significant contributions to clinical cancer therapy due to their great biocompatibility, biodegradability, low toxicity and favorable mechanical or viscoelastic properties. Accordingly, several types of self-assembled hydrogels have been approved for preclinical or clinical trials (Webber and Pashuck, 2021; Li et al., 2022). Through intramolecular covalent or noncovalent interactions between polymers, hydrogels could better control the release of laden drugs in a more sustainable way and prevent their distribution in off-target tissues, which may efficiently alleviate the side effects or toxicity due to high dosages, make chemotherapies more tolerable and thus improve antitumor efficacy (Hu et al., 2021). Among them, owing to the sensitivity to temperature, thermosensitive polymers may undergo sol-gel transition under physiological environment without the participation of organic solvent, thus decreasing the toxicity of these formulations. Together with its injectability, thermosensitive hydrogels are applicable for local delivery of various chemotherapeutics.

**SCHEME 1:**
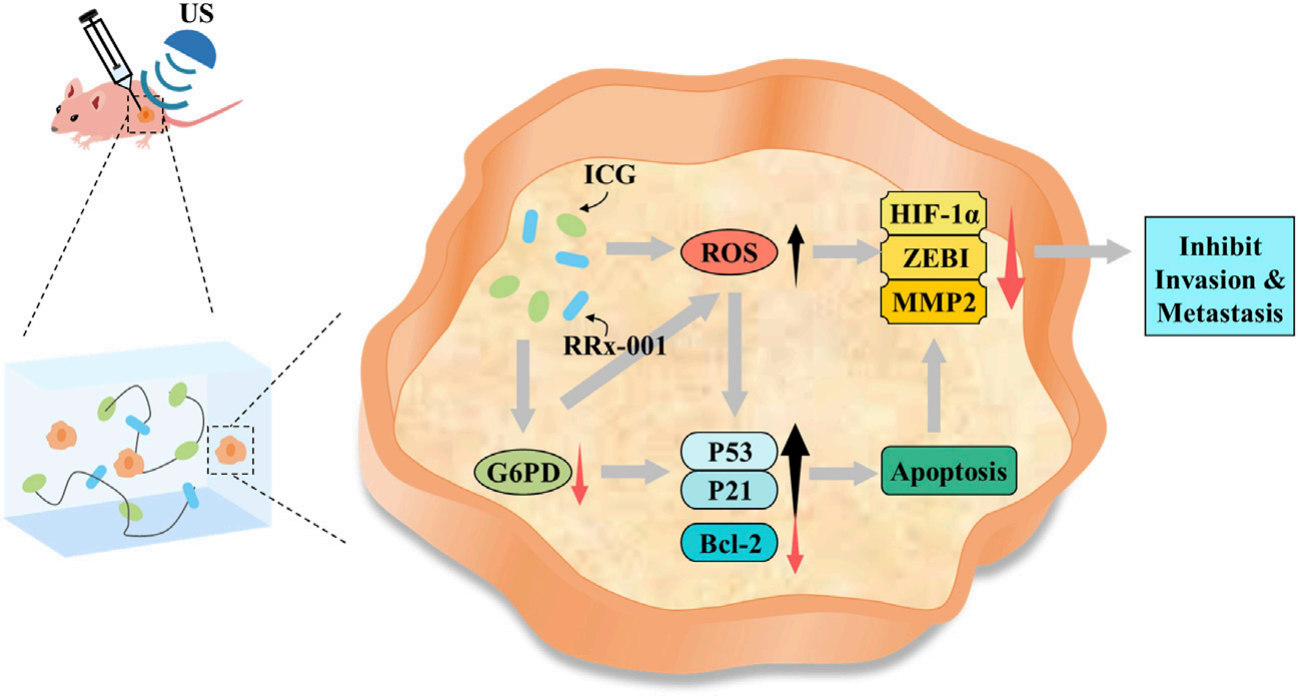
Potential mechanism of IR@CPGel for synergistic breast cancer therapy integrated with LIFU.

Herein, in this study, a thermosensitive chitosan-pluronic copolymer was designed and synthesized for the preparation of self-assembled CPGel for the codelivery of ICG (sonosensitive agent) and RRx-001 as indicated in Scheme 1. MDA-MB-231 (Human Breast Cancer Cell Line) was introduced as a model to investigate the antitumor efficacy of ICG and RRx-001 coloaded CPGgel (IR@PCGel) under LIFU irradiation. The potential antitumor and antimetastatic mechanisms of IR@PCGel were investigated at the histological and molecular levels. Its biocompatibility and toxicity were also studied by monitoring biochemical indices and pathological changes in major organ tissues. Taken together, this work aimed to enhance the efficacy of sonodynamic therapy through local administration of self-assembled IR@CPGel to interfere with redox homeostasis and thus amplify the oxidative stress microenvironment in tumor tissues. In a word, this work provides a new strategy for the SDT enhancement in breast cancer therapy.

## Materials and Methods

### Materials

Chitosan, Pluronic F127,1-ethyl-3-(3-dimethylaminopropyl)-carbodiimide (EDC), N-hydroxysuccinimide (NHS), succinic anhydride, 4-morpholineethanesulfonic acid (MES), 4-dimethylaminopyridine (DMAP), indocyanine green (ICG) and RRx-001 were purchased from Sigma–Aldrich and MCE (United States). Primary antibodies, including anti-G6PD, anti-P21, anti-P53, anti-Bcl-2, anti-MMP2, anti-ZEB1 and anti-HIF-1α, were purchased from Abcam (Cambridge, United Kingdom). The 4′,6-diamidino-2-phenylindole (DAPI), Calcein AM, propidium iodide (PI), 2,7-dichlorodihydrofluorescein diacetate (DCF), TUNEL assay kit and BCA kit were purchased from Thermo Fisher Scientific (United States).

### Synthesis and Characterization of ICG/RRx-001@CPGel

Chitosan-*co*-Pluronic polymer (CP) was synthesized *via* the EDC/NHS reaction as previously reported (Park et al., 2009). For preparation of CP hydrogel or ICG/RRx-001@CPGel (IR@CPGel), the as-synthesized CP polymers were dissolved in PBS buffer at a concentration of 20% wt at 4°C. ICG (0.5 mg/ml) or RRx-001 (1 mg/ml) dispersed in 20% wt CP solution was prepared and kept at 4°C for further use. After that, the mixture was kept in an oven at 37°C to allow sol-gel transition for further characterization by cryogenic scanning electron microscopy (cyro-SEM) (HITACHI, SU 8020, Japan), and the sol-gel transition of copolymer aqueous solutions was detected by MARS60 (HAAKE, Germany).

### Cell Lines and Cell Culture

Cell lines including MDA-MB-231, L929 and HUVECs were purchased from American Type Culture Collection (Manassas, VA) and cultured in DMEM supplemented with 10% fetal bovine serum, 100 units/ml penicillin and 50 units/ml streptomycin at 37°C in a humidified incubator with a 5% CO_2_ atmosphere.

### Detection of ROS Generation

DCF probe was used to detect ROS generation in cells treated with different formulations. At first, MDA-MB-231 cells were seeded in 6-well plates at a density of 2.0 × 10^5^ cells/ml followed by overnight incubation. After that, the cells were treated with different formulations for 2 h: (1) PBS; (2) LIFU (1 W/cm^2^, 1 min) + CPGel; (3) RRx-001@CPGel; (4) LIFU (1 W/cm^2^, 1 min) + RRx-001@CPGel; (5) LIFU (1 W/cm^2^, 1 min) + ICG@CPGel; and (6) LIFU (1 W/cm^2^, 1 min) + IR@CPGel. Medium containing the formulations was then removed and replenished with fresh medium containing DCF probe. After 30 min staining, the DCF probe was removed, and the cells were rinsed with PBS 3 times for further detection with a fluorescence microscope (Leica, DM28, Germany) at *ex:* 485 nm and *em:* 520 nm. ROS generation in cells was then semiquantified by ImageJ software.

### Calcein AM/PI Costaining

Calcein AM/PI costaining was introduced to investigate apoptosis in cells treated with IR@CPGel as previously described (Wang et al., 2022). MDA-MB-231 cells were firstly seeded in 6-well plates at a density of 2.0 × 10^5^ cells/ml followed by overnight incubation. After incubation, the cells were treated as following description for 2 h: (1) PBS; (2) LIFU (1 W/cm^2^, 1 min) + CPGel; (3) RRx-001@CPGel; (4) LIFU (1 W/cm^2^, 1 min) + RRx-001@CPGel; (5) LIFU (1 W/cm^2^, 1 min) + ICG@CPGel; and (6) LIFU (1 W/cm^2^, 1 min) + IR@CPGel. Calcein AM and PI probes were then added to each well and incubated with cells for an hour. Finally, cells were washed with PBS 3 times for further observation under a fluorescence microscope (Leica, DM28, Germany) with *ex:* 495 nm for the detection of live cells and *ex:* 545 nm for the detection of apoptotic cells. The apoptosis rates from different treatment groups were analyzed by ImageJ software.

### *In Vivo* Release of ICG@CPGel

Nude mice were used to investigate the release kinetics of ICG@CPGel. Briefly, nude mice were randomly divided into two groups: 1) ICG: *s. c.* injection of 100 µl ICG; 2) ICG@CPGel: *s. c.* injection of ICG@CPGel (100 µl). The whole evaluation lasted for 5 days, during which ICG signals were monitored by IVIS Spectrum (Perkin Elmer, United States) every day.

### *In Vivo* Antitumor Efficacy of ICG/RRx-001@CPGel

MDA-MB-231 cell xenograft mouse models were established by subcutaneous (*s.c.*) injection of 2 × 10^6^ MDA-MB-231 cells in nude mice until the tumor volume grew to 100 mm^3^. MDA-MB-231 cell xenograft mouse models were then randomly divided into six groups: (1) Control group: intratumoral injection (*i.t.*) of 100 µl saline solution; (2) *i. t.* injection of CPGel (100 µl) + LIFU; (3) *i. t.* injection of RRx-001@CPGel (100 μl, 100 µg RRx-001); (4) *i. t.* injection of RRx-001@CPGel (100 μl, 100 µg RRx-001) + LIFU; (5) *i. t.* injection of ICG@CPGel (100 μl, 50 µg ICG) + LIFU; (6) *i. t.* injection of IR@CPGel (100 μl, 100 µg RRx-001, 50 µg ICG) + LIFU. LIFU was performed on days 0, 2, and 4, and the parameters were performed as follows: power density = 1 W/cm^2^, duty cycle = 50%, transducer = 1 MHz, and duration = 5 min. Different formulations were administered on day 0. Mouse weight and tumor volume were monitored every other day. The whole evaluation lasted for 40 days. After the evaluation, all mice were euthanized, and tumors from all treatment groups were collected for H&E staining, immunofluorescence analysis (MMP-2, ZEB1 and HIF-1α) and TUNEL assay.

### Immunofluorescence Staining

MMP-2, ZEB1 and HIF-1α were all detected by immunofluorescence staining to evaluate the antitumor efficacy of IR@CPGel. In brief, tumor slices were dewaxed and rehydrated first. Then, the slices were immersed in PBST, and antigens were retrieved in a microwave oven for 45 s of heating. After cooling, tumor slices were covered with 3% hydrogen peroxide to inactivate endogenous peroxidase, followed by rinsing with PBST 3 times. After that, 10% goat serum was mounted on tumor slices and blocked for 30 min. After three rinses with PBST, the tumor slices were covered with the corresponding fluorescence-labeled antibodies and incubated at 4°C overnight. The slides were finally washed with PBST three times and mounted for further observation.

### Western Blotting Analysis

Western blotting analysis was further performed to investigate the antitumor mechanism of IR@CPGel. Tumor tissues were homogenized in RIPA buffer at 4°C and extracted for 30 min, followed by centrifugation at 12,000 rpm to collect protein samples. A BCA kit was used to determine the protein concentration of each sample. The expression levels of G6PD, P21, P53 and Bcl-2 were then investigated through western blotting analysis based on our previous protocol. β-actin was used as an internal reference to verify the same loading in each lane. The relative expression of target proteins was analyzed by ImageJ software.

### Biocompatibility and Toxicity of IR@CPGel

Another group of nude mice was used to investigate the biosafety of IR@CPGel. In brief, nude mice were randomly divided into two groups: (1) healthy control: *s. c.* injection of 100 µl saline solution; (2) IR@CPGel: *s. c.* injection of IR@CPGel (100 µl). The whole evaluation lasted for a month. Blood samples and organs from the IR@CPGel group were collected on Day 15 and Day 30, while samples from healthy controls were collected on Day 30.

Collected organs were fixed in 4% polyformaldehyde and immersed in paraffin. The fixed organs were then cut into 4 µm slices for further staining. In brief, organs slices were dewaxed and rehydrated at first and then stained with hemoxylin and eosin for 2–5 min. After staining, organ slices were rinsed with water and rehydrated following with the mount of resin for further observation under optical microscopy.

Serum was obtained from collected blood samples by centrifugation at 3,000 rpm. Levels of biochemical parameters, including total protein (TP), albumin (ALB), globulin (GLB), alanine aminotransferase (ALT), aspartate aminotransferase (AST), uric acid (UA), UREA and creatinine clearance (CCr), in the serum were determined by an automatic biochemical analyzer.

### Statistical Analysis

All values are expressed as the mean ± standard deviation (SD). Comparisons among all groups were evaluated using one-way ANOVA or Student’s t-test by GraphPad Prism version 5.0 for Windows (GraphPad Software, United States), and data with *p* < 0.05, *p* < 0.01, or *p* < 0.001 were considered statistically significant.

## Results and Discussions

### Preparation and Characterization of IR@CPGel

**FIGURE 1:**
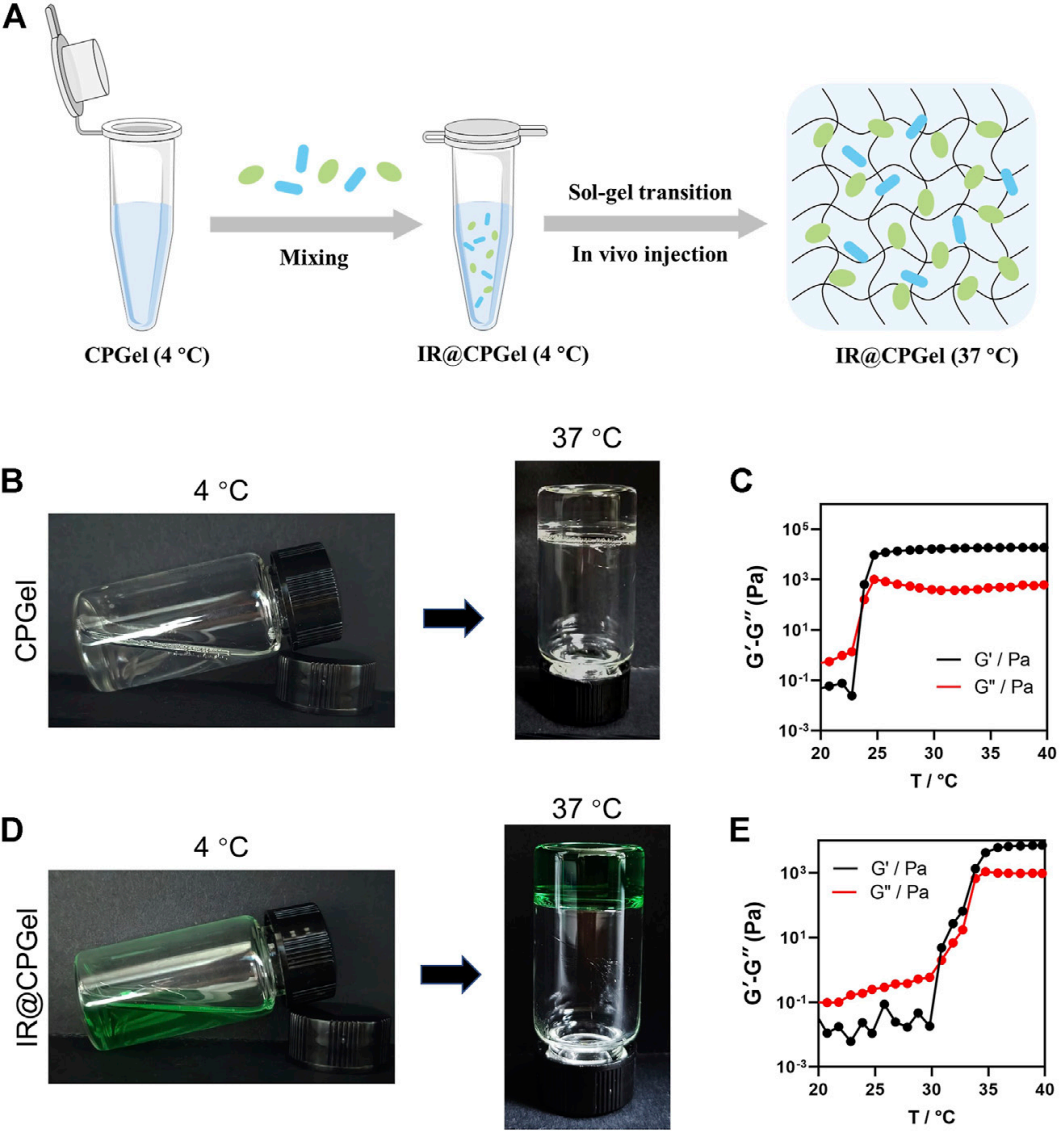
Preparation and characterization of IR@CPGel. **(A)** Schematic illustration of procedures for IR@CPGel preparation; **(B)** representative photos of CPGel undergoing the sol-gel transition; **(C)** dynamic mechanical analysis of CPGel; **(D)** representative photos of IR@CPGel undergoing the sol-gel transition; **(E)** dynamic mechanical analysis of IR@CPGel.

A thermosensitive copolymer, chitosan-pluronic copolymer, was used in this work to construct ICG and RRx-001 coloaded hydrogels (IR@CPGel). Pluronic with polypropylene oxide as the central hydrophobic part and polyethylene oxide (PEO) as the hydrophilic part is a kind of nonionic thermosensitive triblock copolymer that may self-assemble into hydrogels under physiological temperature (37°C). However, its weak mechanical properties and fast clearance rate resulting from rapid degradation under physiological conditions always lead to uncontrollable release kinetics (Nie et al., 2021). Therefore, in this work, chitosan was grafted onto the Pluronic backbone through the EDC/NHS reaction as previously reported. IR@CPGel was then prepared through the self-assembly property of the CP copolymer at 37°C, as indicated in Figure 1A. A rotational rheometer was then used to investigate whether the addition of ICG and RRx-001 may change the lower critical solution temperature (LCST) of CPGel. As shown in Figures 1B–E, both CPGel and IR@CPGel could realize the sol-gel transition at 37°C, confirming the thermosensitive property of CP and ensuring the sol-gel transition at physiological temperature. More specifically, CPGel without the addition of drugs exhibited a relatively low LCST (∼25°C), while in IR@CPGel, the LCST was approximately 30°C. The morphology of CPGel was then further investigated by SEM, as indicated in Supplementary Figure S1. CPGel exhibited a 3D porous structure with a pore diameter of ∼100 μm, ensuring the localized and sustained delivery of ICG and RRx-001. Therefore, the release kinetics of ICG@CPGel were investigated in nude mice, as indicated in Supplementary Figure S2. It could obviously be noted that ICG@CPGel could effectively maintain the release of ICG for more than 5 days. On Day 5, a high fluorescence signal of ICG could still be detected at the injection site, demonstrating that the half-life and release phase of the laden drugs were efficiently prolonged. Taken together, this self-assembled IR@CPGel holds the potential to be investigated further *in vitro* and *in vivo*.

### *In Vitro* Antitumor Efficacy of IR@CPGel

**FIGURE 2:**
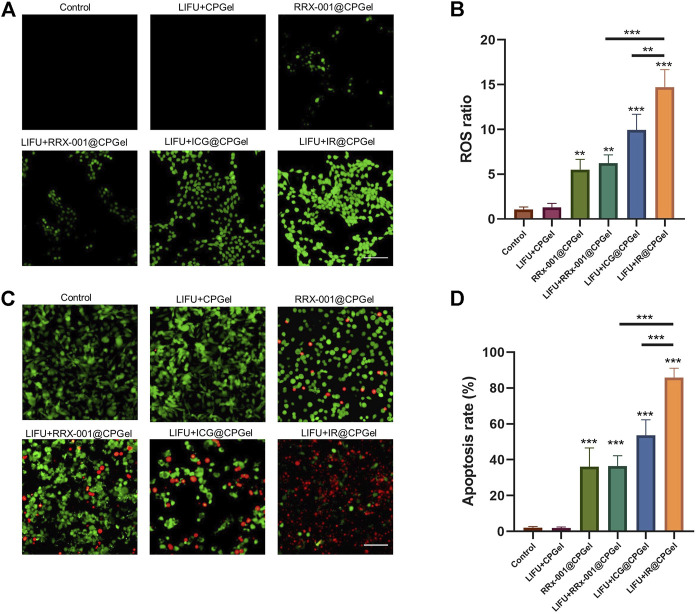
*In vitro* antitumor efficacy of IR@CPGel. **(A)** ROS level induced by different formulations in MDA-MB-231 cells; scale bar = 100 µm **(B)** Semiquantitative analysis of ROS levels from different treatment groups; **(C)** Calcein-AM/PI staining of MDA-MB-231 cells treated with different formulations; scale bar = 100 µm **(D)** Semiquantitative analysis of apoptosis rate in MDA-MB-231 cells treated with different formulations. All data are shown as the mean ± SD. (n = 3), ∗∗*p* < 0.01 and ∗∗∗*p* < 0.001 vs. Control.

Oxidative stress plays a critical role in cell apoptosis, especially the intrinsic pathway. Combined with new emerging sonodynamic therapies applied in breast cancer treatment, the antitumor efficacy of IR@CPGel was first studied. Therefore, ROS level in MDA-MB-231 cells treated with different formulations was detected. As shown in Figures 2A,B, without the addition of ICG, the ROS level in the LIFU + CPGel group did not increase obviously, which was comparable with the control group and thus proved the low harm of LIFU to cells or tissues. In the LIFU + ICG@CPGel group, the ROS ratio was enhanced to ∼9, demonstrating that ICG may trigger significant ROS generation under LIFU irradiation. In the LIFU + IR@CPGel group, the ROS ratio was even enhanced to ∼14, which may be due to the synergistic effect of ICG and RRx-001. Intriguingly, in cells treated with RRx-001@CPGel or LIFU + RRx-001@CPGel, the ROS ratio (∼5) did not increase greatly, and irradiation with LIFU did not enhance the ROS level, indicating that the ROS generated in both groups were induced by RRx-001 without the influence of LIFU.

The apoptosis induced by all these formulations was then investigated by calcein AM/PI costaining. Corresponding with the ROS results, apoptotic cells, represented by red fluorescence, could barely be detected in cells treated with LIFU + CPGel. In cells treated with LIFU + ICG@CPGel or IR@CPGel, the apoptosis rate reached 55 and 85%, respectively. In addition, in the RRx-001@CPGel group or LIFU + RRx-001@CPGel group, although the apoptosis rate was only approximately 35%, the antitumor efficacy of RRx-001 was still verified. Therefore, it could be preliminarily deduced that under LIFU irradiation, IR@CPGel could efficiently trigger the overgeneration of ROS in cells and thus induce the apoptosis of cells in a synergistic manner with RRx-001.

### *In Vivo* Antitumor Efficacy of IR@CPGel

**FIGURE 3:**
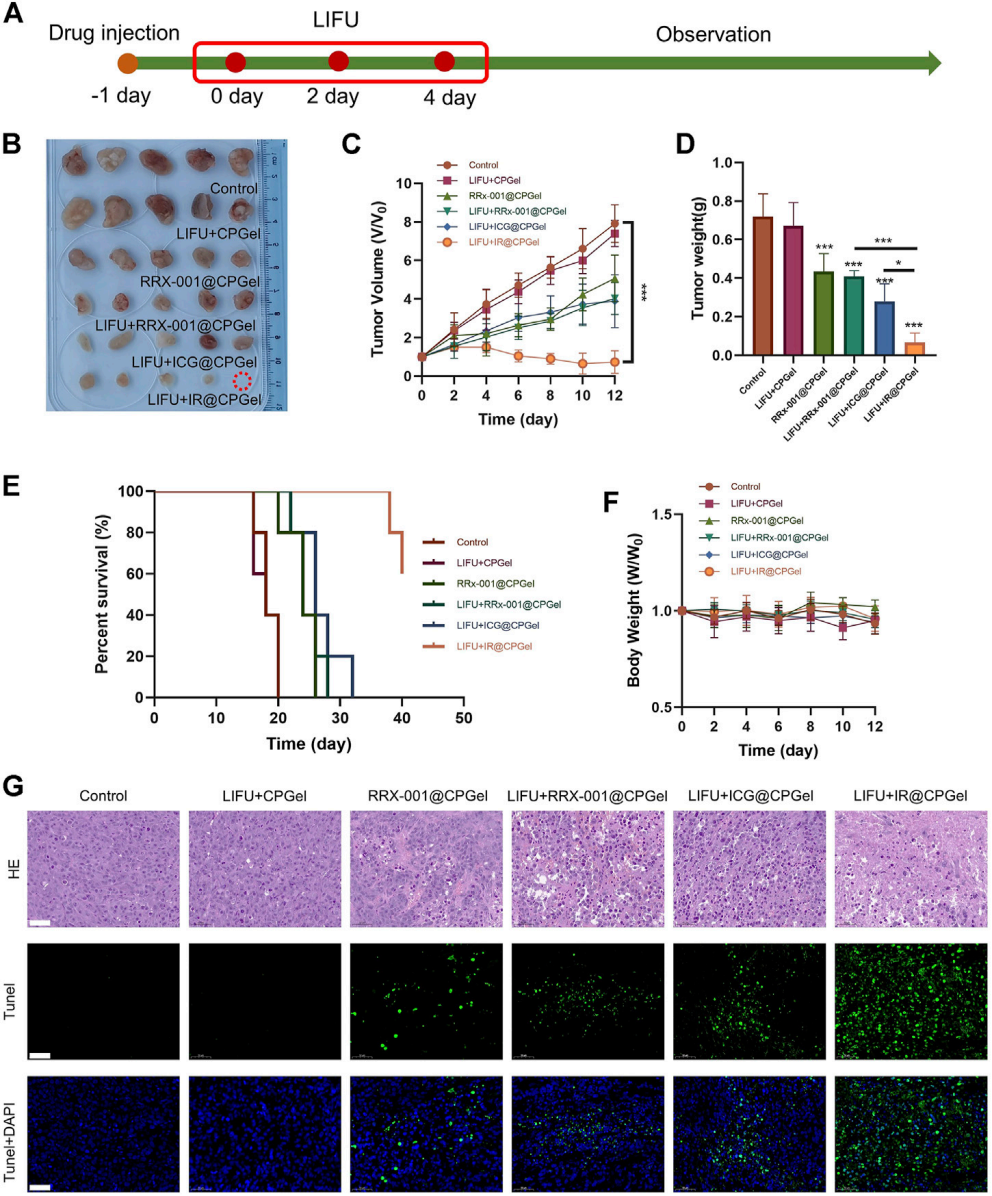
*In vivo* antitumor efficacy of IR@CPGel. **(A)** Schematic diagram of the animal experiment; **(B)** Phots of resected tumors collected from different treatment groups **(C)** Plot of tumor volume vs. time from different treatment groups; **(D)** Tumor weight resected from different treatment groups; **(E)** Survival rate of mice from different treatment groups; **(F)** Body weights of mice from different treatment groups; **(G)** Histological analysis including H&E staining and TUNEL assay of tumor tissues from different treatment groups, scale bar = 50 µm. All data are shown as the mean ± SD. (n = 5), ∗*p* < 0.05 and ∗∗∗*p* < 0.001 vs. Control.

MDA-MB-231 cell xenograft mouse models were used to further evaluate the antitumor efficacy of IR@CPGel as described in Figure 3A. After 12 days of evaluation, as shown in Figures 3B,C, in contrast with the control group, the relative tumor volume in the LIFU + CPGel group did not change greatly, further proving the safety of LIFU and low antitumor efficacy of LIFU without the participant of sono-sensitizer. Moreover, the resected relative tumor volume from the LIFU + IR@CPGel group was significantly decreased (***p < 0.001) by more than 87.5% compared with that of the control group. Additionally, the other treatment groups could also effectively inhibit tumor growth and maintain a relative tumor volume of ∼3. Mouse tumor weight from different treatment groups (Figure 3D) further confirmed our findings, compare to the resected tumor weight from control group, tumor weight from LIFU + RRx-001@CPGel group, LIFU + ICG@CPGel group and LIFU + IR@CPGel group were decreased by approximately 42.8, 57 and 85.7% respectively. Together with the survival rate (Figure 3E), it could be concluded that LIFU + IR@CPGel exhibited the best antitumor efficacy since after 40 days of monitoring, the survival rate of mice from the LIFU + IR@CPGel group could still be up to 60%. Moreover, during the treatment, the mouse body weights from all treatment groups (Figure 3F) did not decrease significantly compared with the control group, indicating the great biocompatibility and low toxicity of IR@CPGel preliminarily.

The antitumor efficacy of IR@CPGel was further verified in terms of histology. As observed in Figure 3G, in tumor slices stained by H&E, the proliferation of cancer cells was effectively inhibited, as the purple color was significantly decreased in the LIFU + IR@CPGel group. The TUNEL assay and its semi-quantitative analysis (Supplementary Figure S3) further verified the apoptosis of cancer cells since in the LIFU + IR@CPGel group, the intensity of green fluorescence representing the fragments of DNA was higher than that from the control group and the other treatment groups. Taken together, these results demonstrated that when integrated with LIFU therapy, IR@CPGel could induce the apoptosis of cancer cells and thus effectively inhibit tumor growth in a safe manner, which may hold the potency to be applied in the clinical therapy of breast cancer.

### Antitumor Mechanism of IR@CPGel

**FIGURE 4:**
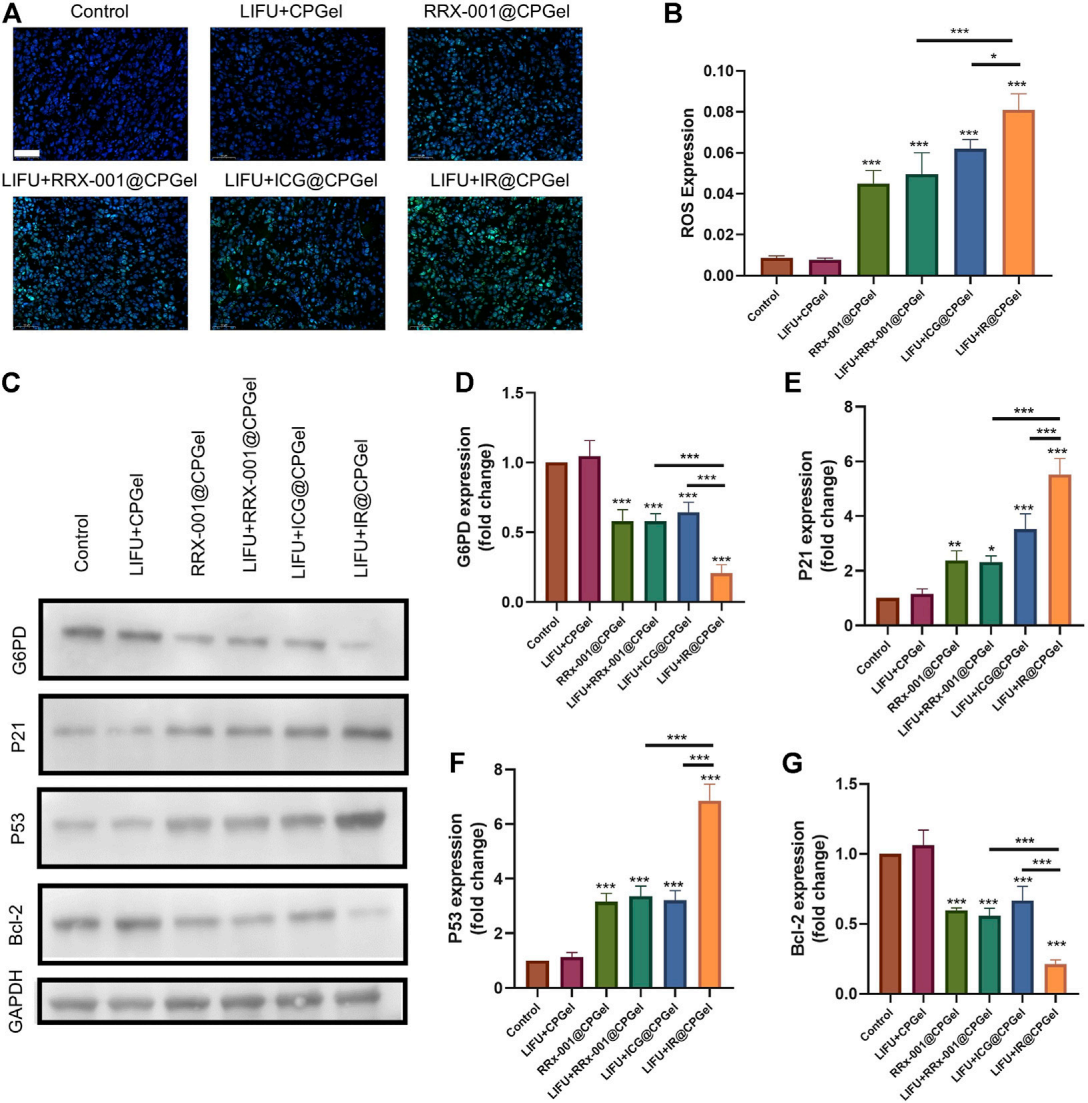
Antitumor mechanism of IR@CPGel. **(A)** ROS levels in mouse tumors treated with different formulations; scale bar = 50 µm. **(B)** Semiquantitative analysis of ROS levels in mouse tumors treated with different formulations. **(C)** Western blotting analysis of G6PD, P21, P53 and Bcl-2 expression levels in mouse tumors treated with different formulations. Semiquantitative analysis of **(D)** G6PD, **(E)** P21, **(F)** P53, and **(G)** Bcl-2 expression in mouse tumors treated with different formulations. All data are shown as the mean ± SD. (n = 3), ∗*p* < 0.05, ∗∗*p* < 0.01 and ∗∗∗*p* < 0.001 vs. Control.

After confirming its antitumor efficacy both in cells and mice, the antitumor mechanism of IR@CPGel was further investigated and clarified by immunofluorescence analysis and western blotting analysis. ROS levels in tumor tissues were first detected, as shown in Figures 4A,B. Similar to the results in cells, ROS signals (green fluorescence) in tumor tissues from the LIFU + IR@CPGel group was significantly stronger than that from the LIFU + ICG@CPGel group (*p < 0.05) or LIFU + RRx-001@CPGel group (***p < 0.001). These results proved that irradiation with LIFU could efficiently penetrate tumor tissues and trigger the overgeneration of ROS to induce cell apoptosis.

The synergistic mechanism between ICG and RRx-001 was further studied. As shown in Figures 4C,D, G6PD expression from the LIFU + RRx-001@CPGel group or RRx-001@CPGel group was lower than that from the LIFU + ICG@CPGel group, suggesting the potential inhibitive effect of RRx-001 on G6PD expression. This inhibition effect was even obvious in the LIFU + IR@CPGel group. To date, G6PD, as a gatekeeper enzyme, is critical for redox homeostasis and the adaptation of cancer cells against oxidative stress. The downregulation of G6PD would consequently influence the expression of apoptosis-related proteins. Therefore, we also detected the expression of P21, P53, and Bcl-2 in tumor tissues. As shown in Figures 4E–G, proapoptotic signals, including P21 and P53, were enhanced. Moreover, P21 and P53 expression in the LIFU + IR@CPGel group was significantly upregulated than that in the LIFU + RRx-001@CPGel group (***p < 0.001) or LIFU + ICG@CPGel group (***p < 0.001) either. Correspondingly, Bcl-2 expression in the LIFU + IR@CPGel group was significantly reduced compared to that in the LIFU + ICG@CPGel group or LIFU + RRx-001@CPGel group.

**FIGURE 5:**
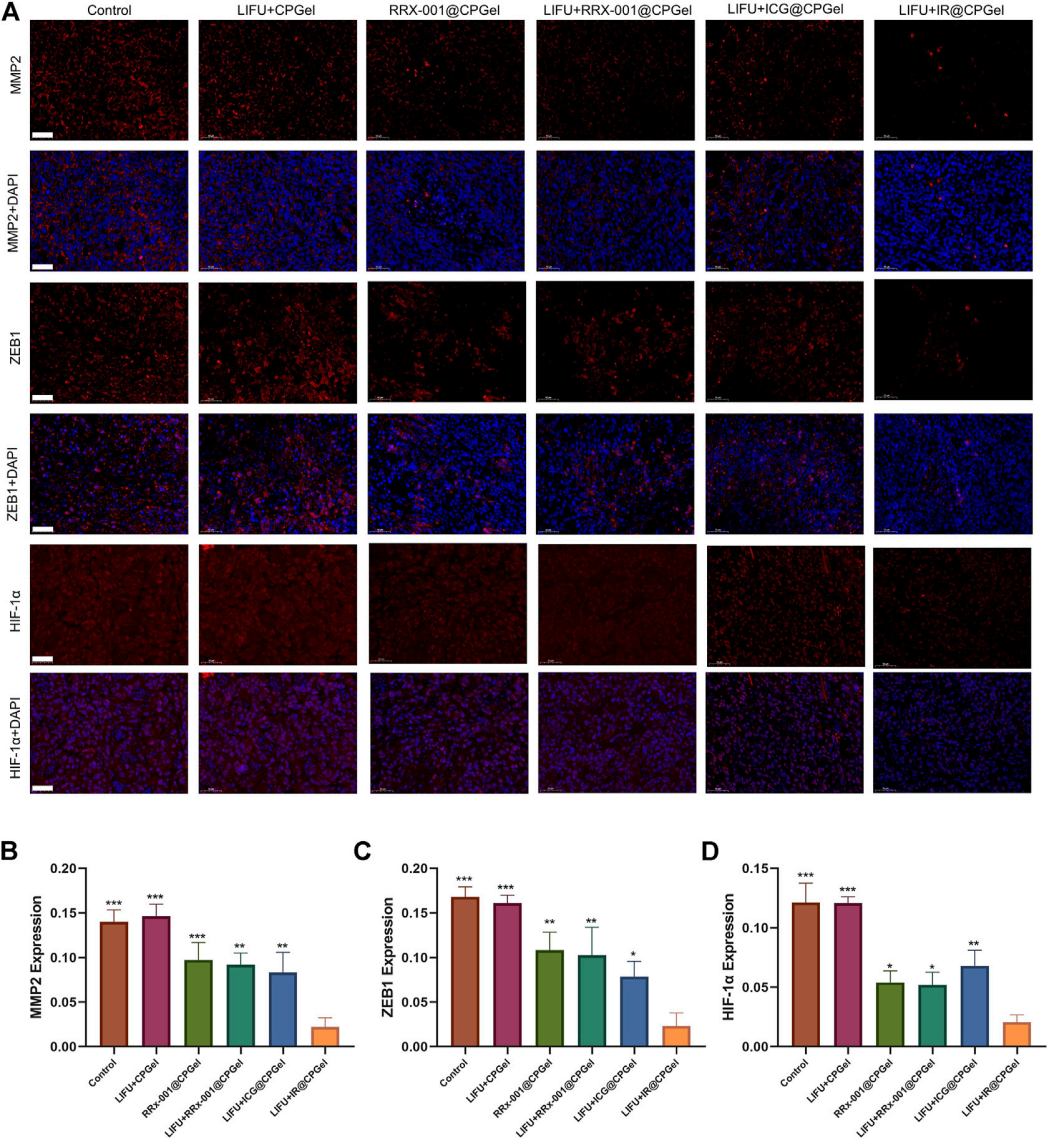
**(A)** Immunofluorescence analysis of MMP-2, ZEB1 and HIF-1α expression levels in mouse tumors treated with different formulations; scale bar = 50 µm. Semiquantitative analysis of MMP-2, ZEB1 and HIF-1α expression levels in mouse tumors treated with different formulations. All data are shown as the mean ± SD. (n = 3), ∗*p* < 0.05, ∗∗*p* < 0.01 and ∗∗∗*p* < 0.001 vs. the LIFU + IR@CPGel group.

The antimetastatic potential of IR@CPGel was also investigated. As shown in Figures 5A–C, MMP-2 and ZEB1 expression in the LIFU + IR@CPGel group was significantly downregulated compared to the other treatment groups. Remarkably, in Figures 5A,D, HIF-1α expression from the LIFU + RRx-001@CPGel group or RRx-001@CPGel group was ∼10% lower than that from the LIFU + ICG@CPGel group. This is mainly due to the downregulation of G6PD induced by RRx-001 interfering with the production of NADPH, a critical enzyme for redox homeostasis. Codelivery of ICG and RRx-001 through self-assembled CPGel significantly reduced metastasis-related proteins to a greater extent.

Based on all these results, we speculate that when integrated with LIFU irradiation, self-assembled IR@CPGel exhibited antitumor efficacy mainly by enhancing ROS generation and thus triggering the intrinsic apoptotic pathway of cancer cells. Moreover, with G6PD as the regulatory target, RRx-001 may interfere with redox hemostasis in cancer cells, promote more ROS production and thus induce oxidative stress. Due to this redox imbalance, proapoptotic signals, such as P21 and P53, were enhanced, and metastasis-related signals, including MMP-2, ZEB1 and HIF-1α, were effectively reduced. In summary, combined with sonodynamic therapy, local administration of self-assembled IR@CPGel can effectively inhibit the proliferation and metastasis of tumor cells, which is applicable in the SDT of breast cancer clinically.

### Biocompatibility and Toxicity of IR@CPGel

The biocompatibility and toxicity of hydrogels are vital for their further clinical translation. Cytotoxicity was firstly investigated on L929 and HUVEC cells as shown in Supplementary Figure S4. After confirming its low cytotoxicity, we also monitored the biochemical indices and pathological changes in major tissues for 30 days to further investigate the biosafety of IR@CPGel. As seen from Supplementary Figures S5, S6, *s. c.* injection of IR@CPGel did not induce obvious pathological changes in mouse major organs, including the lung, heart, spleen, liver, kidney and skin. In addition, biochemical indices (TP, ALB, GLB, ALT, AST, urea, CCr, UA) indicating liver and kidney function were all in the normal range compared to the healthy control group, demonstrating that the local administration of IR@CPGel did not induce any acute toxicity to either the liver or kidney.

## Conclusion

In this study, on the basis of a thermosensitive chitosan-pluronic copolymer, self-assembled CPGel was prepared at physiological temperature (37°C) and successfully loaded with ICG (sonosensitive agent) and RRx-001. Both *in vitro* and *in vivo* antitumor investigations verified that when integrated with sonodynamic therapy applied in breast cancer treatment, local administration of IR@CPgel could enhance ROS generation under LIFU irradiation and trigger the intrinsic apoptotic pathway of cancer cells, thus effectively inhibiting tumor growth in a safe manner. Moreover, with G6PD as the regulatory target, RRx-001 may interfere with redox hemostasis in cancer cells, promote more ROS production and thus induce oxidative stress. Due to this redox imbalance, proapoptotic signals, such as P21 and P53, were enhanced, and metastasis-related signals, including MMP-2, ZEB1 and HIF-1α, were effectively reduced. Together with its high biocompatibility and low toxicity, local administration of such self-assembled IR@CPGel may hold great potency to enhance the SDT efficiency in clinical breast cancer therapy.

## Data Availability

The raw data supporting the conclusions of this article will be made available by the authors, without undue reservation.

## References

[B1] Azamjah N. Soltan-Zadeh Y. Zayeri F. (2019). Global Trend of Breast Cancer Mortality Rate: a 25-year Study. Asian Pac J. Cancer Prev. 20, 2015–2020. 10.31557/apjcp.2019.20.7.2015 31350959 PMC6745227

[B2] Carioli G. Malvezzi M. Rodriguez T. Bertuccio P. Negri E. La Vecchia C. (2017). Trends and Predictions to 2020 in Breast Cancer Mortality in Europe. Breast 36, 89–95. 10.1016/j.breast.2017.06.003 28988610

[B3] Catanzaro D. Gaude E. Orso G. Giordano C. Guzzo G. Rasola A. (2015). Inhibition of Glucose-6-Phosphate Dehydrogenase Sensitizes Cisplatin-Resistant Cells to Death. Oncotarget 6, 30102–30114. 10.18632/oncotarget.4945 26337086 PMC4745784

[B4] Cho E. S. Cha Y. H. Kim H. S. Kim N. H. Yook J. I. (2018). The Pentose Phosphate Pathway as a Potential Target for Cancer Therapy. Biomol. Ther. 26, 29–38. 10.4062/biomolther.2017.179 PMC5746035 29212304

[B5] Costley D. Mc Ewan C. Fowley C. Mchale A. P. Atchison J. Nomikou N. (2015). Treating Cancer with Sonodynamic Therapy: a Review. Int. J. Hyperth. 31, 107–117. 10.3109/02656736.2014.992484 25582025

[B6] Das D. S. Ray A. Das A. Song Y. Tian Z. Oronsky B. (2016). A Novel Hypoxia-Selective Epigenetic Agent RRx-001 Triggers Apoptosis and Overcomes Drug Resistance in Multiple Myeloma Cells. Leukemia 30, 2187–2197. 10.1038/leu.2016.96 27118403 PMC5093055

[B7] Fu J. Li T. Zhu Y. Hao Y. (2019). Ultrasound‐Activated Oxygen and ROS Generation Nanosystem Systematically Modulates Tumor Microenvironment and Sensitizes Sonodynamic Therapy for Hypoxic Solid Tumors. Adv. Funct. Mat. 29, 1906195. 10.1002/adfm.201906195

[B8] He L. He T. Farrar S. Ji L. Liu T. Ma X. (2017). Antioxidants Maintain Cellular Redox Homeostasis by Elimination of Reactive Oxygen Species. Cell. Physiol. Biochem. 44, 532–553. 10.1159/000485089 29145191

[B9] He L. Nie T. Xia X. Liu T. Huang Y. Wang X. (2019). Designing Bioinspired 2D MoSe 2 Nanosheet for Efficient Photothermal‐Triggered Cancer Immunotherapy with Reprogramming Tumor‐Associated Macrophages. Adv. Funct. Mat. 29, 1901240. 10.1002/adfm.201901240

[B10] Hu H. Chen J. Yang H. Huang X. Wu H. Wu Y. (2019). Potentiating Photodynamic Therapy of ICG-Loaded Nanoparticles by Depleting GSH with PEITC. Nanoscale 11, 6384–6393. 10.1039/c9nr01306g 30888375

[B11] Hu K. Xie L. Zhang Y. Hanyu M. Yang Z. Nagatsu K. (2020). Marriage of Black Phosphorus and Cu2+ as Effective Photothermal Agents for PET-Guided Combination Cancer Therapy. Nat. Commun. 11, 2778. 10.1038/s41467-020-16513-0 32513979 PMC7280494

[B12] Hu K. Wu W. Xie L. Geng H. Zhang Y. Hanyu M. (2021). Whole-body PET Tracking of a D-Dodecapeptide and its Radiotheranostic Potential for PD-L1 Overexpressing Tumors. Acta Pharm. Sin. B 12, 1363–1376. 10.1016/j.apsb.2021.09.016 35530129 PMC9069398

[B13] Kim M. M. Parmar H. Cao Y. Pramanik P. Schipper M. Hayman J. (2016). Whole Brain Radiotherapy and RRx-001: Two Partial Responses in Radioresistant Melanoma Brain Metastases from a Phase I/II Clinical Trial. Transl. Oncol. 9, 108–113. 10.1016/j.tranon.2015.12.003 27084426 PMC4833892

[B14] Kuroki M. Hachimine K. Abe H. Shibaguchi H. Kuroki M. Maekawa S. (2007). Sonodynamic Therapy of Cancer Using Novel Sonosensitizers. Anticancer Res. 27, 3673–3677. 17970027

[B15] Li T. Lu X.-M. Zhang M.-R. Hu K. Li Z. (2022). Peptide-based Nanomaterials: Self-Assembly, Properties and Applications. Bioact. Mater. 11, 268–282. 10.1016/j.bioactmat.2021.09.029 34977431 PMC8668426

[B16] Lin L.-S. Song J. Song L. Ke K. Liu Y. Zhou Z. (2018). Simultaneous Fenton-like Ion Delivery and Glutathione Depletion by MnO2 -Based Nanoagent to Enhance Chemodynamic Therapy. Angew. Chem. 130, 4996–5000. 10.1002/ange.201712027 29488312

[B17] Matés J. M. Campos-Sandoval J. A. de los Santos-Jiménez J. Márquez J. (2020). Glutaminases Regulate Glutathione and Oxidative Stress in Cancer. Arch. Toxicol. 94, 2603–2623. 10.1007/s00204-020-02838-8 32681190

[B18] Nie T. Wang W. Liu X. Wang Y. Li K. Song X. (2021). Sustained Release Systems for Delivery of Therapeutic Peptide/Protein. Biomacromolecules 22, 2299–2324. 10.1021/acs.biomac.1c00160 33957752

[B19] Ning S. Sekar T. V. Scicinski J. Oronsky B. Peehl D. M. Knox S. J. (2015). Nrf2 Activity as a Potential Biomarker for the Pan-Epigenetic Anticancer Agent, RRx-001. Oncotarget 6, 21547–21556. 10.18632/oncotarget.4249 26280276 PMC4673285

[B20] Niu B. Liao K. Zhou Y. Wen T. Quan G. Pan X. (2021). Application of Glutathione Depletion in Cancer Therapy: Enhanced ROS-Based Therapy, Ferroptosis, and Chemotherapy. Biomaterials 277, 121110. 10.1016/j.biomaterials.2021.121110 34482088

[B21] Oronsky B. Scicinski J. Reid T. Oronsky A. Carter C. Oronsky N. (2016). RRx-001, a Novel Clinical-Stage Chemosensitizer, Radiosensitizer, and Immunosensitizer, Inhibits Glucose 6-phosphate Dehydrogenase in Human Tumor Cells. Discov. Med. 21, 251–265. 27232511

[B22] Oronsky B. Paulmurugan R. Foygel K. Scicinski J. Knox S. J. Peehl D. (2017). RRx-001: a Systemically Non-toxic M2-To-M1 Macrophage Stimulating and Prosensitizing Agent in Phase II Clinical Trials. Expert Opin. investigational drugs 26, 109–119. 10.1080/13543784.2017.1268600 27935336

[B23] Park K. M. Lee S. Y. Joung Y. K. Na J. S. Lee M. C. Park K. D. (2009). Thermosensitive Chitosan-Pluronic Hydrogel as an Injectable Cell Delivery Carrier for Cartilage Regeneration. Acta biomater. 5, 1956–1965. 10.1016/j.actbio.2009.01.040 19261553

[B24] Reid T. Oronsky B. Scicinski J. Scribner C. L. Knox S. J. Ning S. (2015). Safety and Activity of RRx-001 in Patients with Advanced Cancer: a First-In-Human, Open-Label, Dose-Escalation Phase 1 Study. Lancet Oncol. 16, 1133–1142. 10.1016/s1470-2045(15)00089-3 26296952

[B25] Sun Y. Zheng Y. Wang C. Liu Y. (2018). Glutathione Depletion Induces Ferroptosis, Autophagy, and Premature Cell Senescence in Retinal Pigment Epithelial Cells. Cell. Death Dis. 9, 753. 10.1038/s41419-018-0794-4 29988039 PMC6037763

[B26] Tang H.-Y. Ho H.-Y. Wu P.-R. Chen S.-H. Kuypers F. A. Cheng M.-L. (2015). Inability to Maintain GSH Pool in G6PD-Deficient Red Cells Causes Futile AMPK Activation and Irreversible Metabolic Disturbance. Antioxidants redox Signal. 22, 744–759. 10.1089/ars.2014.6142 PMC4361223 25556665

[B27] Traverso N. Ricciarelli R. Nitti M. Marengo B. Furfaro A. L. Pronzato M. A. (2013). Role of Glutathione in Cancer Progression and Chemoresistance. Oxidative Med. Cell. Longev. 2013, 1–10. 10.1155/2013/972913 PMC3673338 23766865

[B28] Wang D. Zhou J. Fang W. Huang C. Chen Z. Fan M. (2022). A Multifunctional Nanotheranostic Agent Potentiates Erlotinib to EGFR Wild-type Non-small Cell Lung Cancer. Bioact. Mater. 13, 312–323. 10.1016/j.bioactmat.2021.10.046 35224311 PMC8844835

[B29] Wang F. Shu X. Meszoely I. Pal T. Mayer I. A. Yu Z. (2019). Overall Mortality after Diagnosis of Breast Cancer in Men vs Women. JAMA Oncol. 5, 1589–1596. 10.1001/jamaoncol.2019.2803 31536134 PMC6753503

[B30] Wang X. Zhong X. Gong F. Chao Y. Cheng L. (2020). Newly Developed Strategies for Improving Sonodynamic Therapy. Mat. Horiz. 7, 2028–2046. 10.1039/d0mh00613k

[B31] Webber M. J. Pashuck E. T. (2021). (Macro)molecular Self-Assembly for Hydrogel Drug Delivery. Adv. Drug Deliv. Rev. 172, 275–295. 10.1016/j.addr.2021.01.006 33450330 PMC8107146

[B32] Xing X. Zhao S. Xu T. Huang L. Zhang Y. Lan M. (2021). Advances and Perspectives in Organic Sonosensitizers for Sonodynamic Therapy. Coord. Chem. Rev. 445, 214087. 10.1016/j.ccr.2021.214087

[B33] Yamawaki K. Mori Y. Sakai H. Kanda Y. Shiokawa D. Ueda H. (2021). Integrative Analyses of Gene Expression and Chemosensitivity of Patient-Derived Ovarian Cancer Spheroids Link G6PD-Driven Redox Metabolism to Cisplatin Chemoresistance. Cancer Lett. 521, 29–38. 10.1016/j.canlet.2021.08.018 34419499

[B34] Zhang Y. Zhang X. Yang H. Yu L. Xu Y. Sharma A. (2021). Advanced Biotechnology-Assisted Precise Sonodynamic Therapy. Chem. Soc. Rev. 50, 11227–11248. 10.1039/d1cs00403d 34661214

[B35] Zhao H. Ning S. Scicinski J. Oronsky B. Knox S. J. Peehl D. M. (2015). Epigenetic Effects of RRx-001: a Possible Unifying Mechanism of Anticancer Activity. Oncotarget 6, 43172–43181. 10.18632/oncotarget.6526 26657731 PMC4791224

