# Supplementary material for: Interference With Redox Homeostasis Through a G6PD-Targeting Self-Assembled Hydrogel for the Enhancement of Sonodynamic Therapy in Breast Cancer
Source: Front Chem. 2022 May 4;10:908892. doi: 10.3389/fchem.2022.908892 (PMC9114499; doi:10.3389/fchem.2022.908892)
Supplement: Supplementary file 1 [file DataSheet1.docx]

*Supporting information*

**Interference with Redox Homeostasis through a G6PD-targeting Self-assembled Hydrogel for the Enhancement of Sonodynamic Therapy in Breast Cancer**

Cuiqing Huang^1,2^#, Yuan Xu^1,3^#, Duo Wang^1,3^#, Zerong Chen^1,3^, Weimin Fang^1,3^, Changzheng Shi^1,3^*, Zeyu Xiao^1^*, Liangping Luo^1,3^*

***To whom correspondence should be addressed:**

^1^The Guangzhou Key Laboratory of Molecular and Functional Imaging for Clinical Translation, The First Affiliated Hospital of Jinan University, Guangzhou, China

^2^ Department of Ultrasound, Guangdong Women and Children Hospital, Guangzhou, China

^3^Department of Ultrasound, Guangdong Women and Children Hospital, Guangzhou, China The Medical Imaging Center, The First Affiliated Hospital of Jinan University, Guangzhou, China

***To whom correspondence should be addressed:**

Changzheng Shi: sczcn@126.com

Zeyu Xiao: zeyuxiao@jnu.edu.cn

Liangping Luo: [tluolp@jnu.edu.cn](mailto:tluolp@jnu.edu.cn)


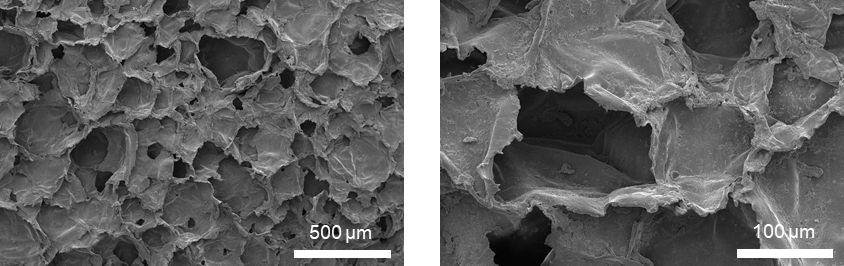


**FIGURE S1** Morphological study of CPGel by SEM.


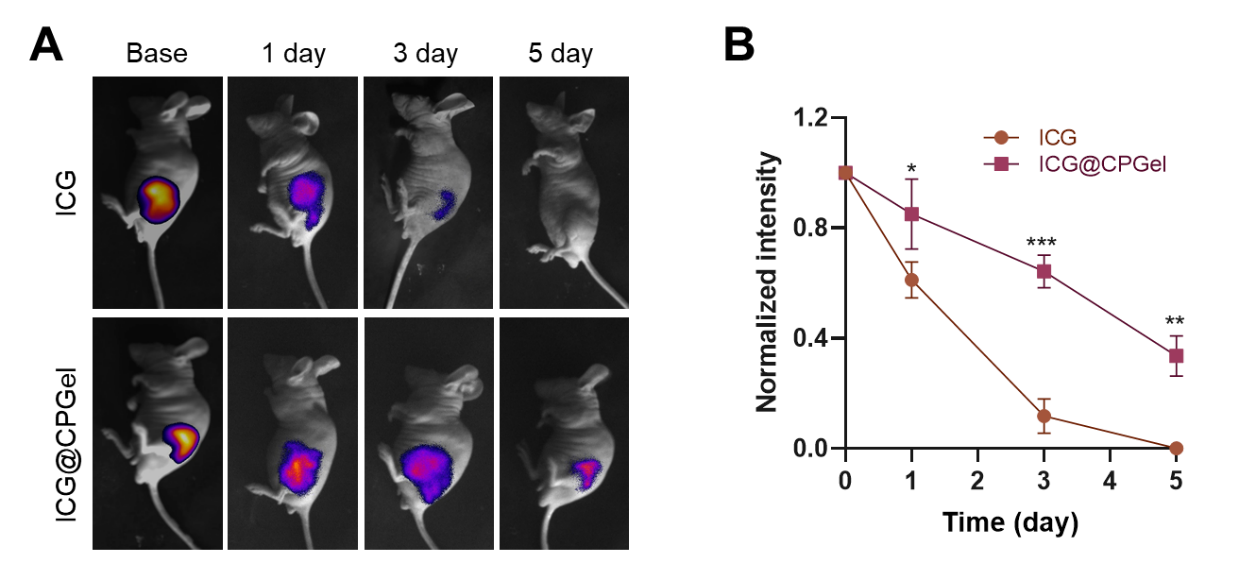


**FIGURE S2** *In vivo* release kinetics of ICG@CPGel analyzed by IVIS Spectrum (Perkin Elmer, USA). (A) Fluorescence imaging of mice after subcutaneous injection of ICG or ICG@CPGel (100 µL); (B) Semi-quantitative analysis of the fluorescence imaging. All data are shown as mean ± S.D. (n = 3). **p* < 0.05, ** *p* < 0.01 and ****p* < 0.001 vs. ICG group.

**FIGURE S3** Semiquantitative analysis of TUNEL assay. All data are shown as mean ± S.D. (n = 3). ***p* < 0.01 and ****p* < 0.001 vs.Control.

**FIGURE S4** Cytotoxicity of CPGel on different cell lines. All data are shown as mean ± S.D. (n = 3).


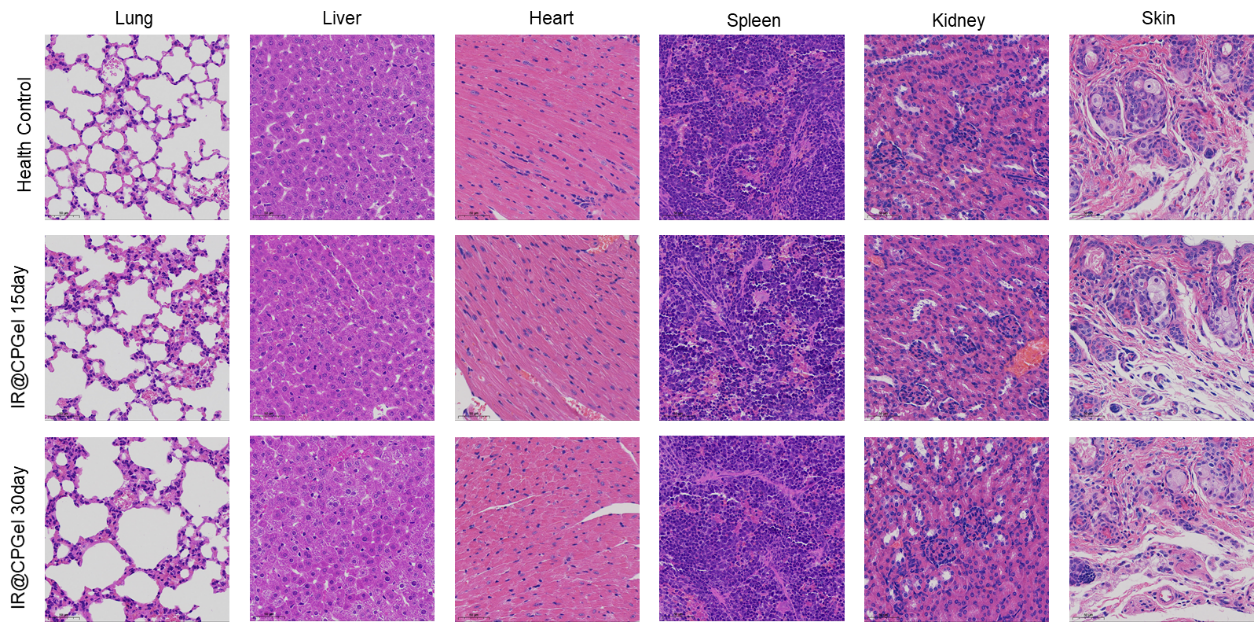


**FIGURE S5** Pathological changes of mice major organs in 30 days after subcutaneous injection of IR@CPGel, scale bar = 50 µm.


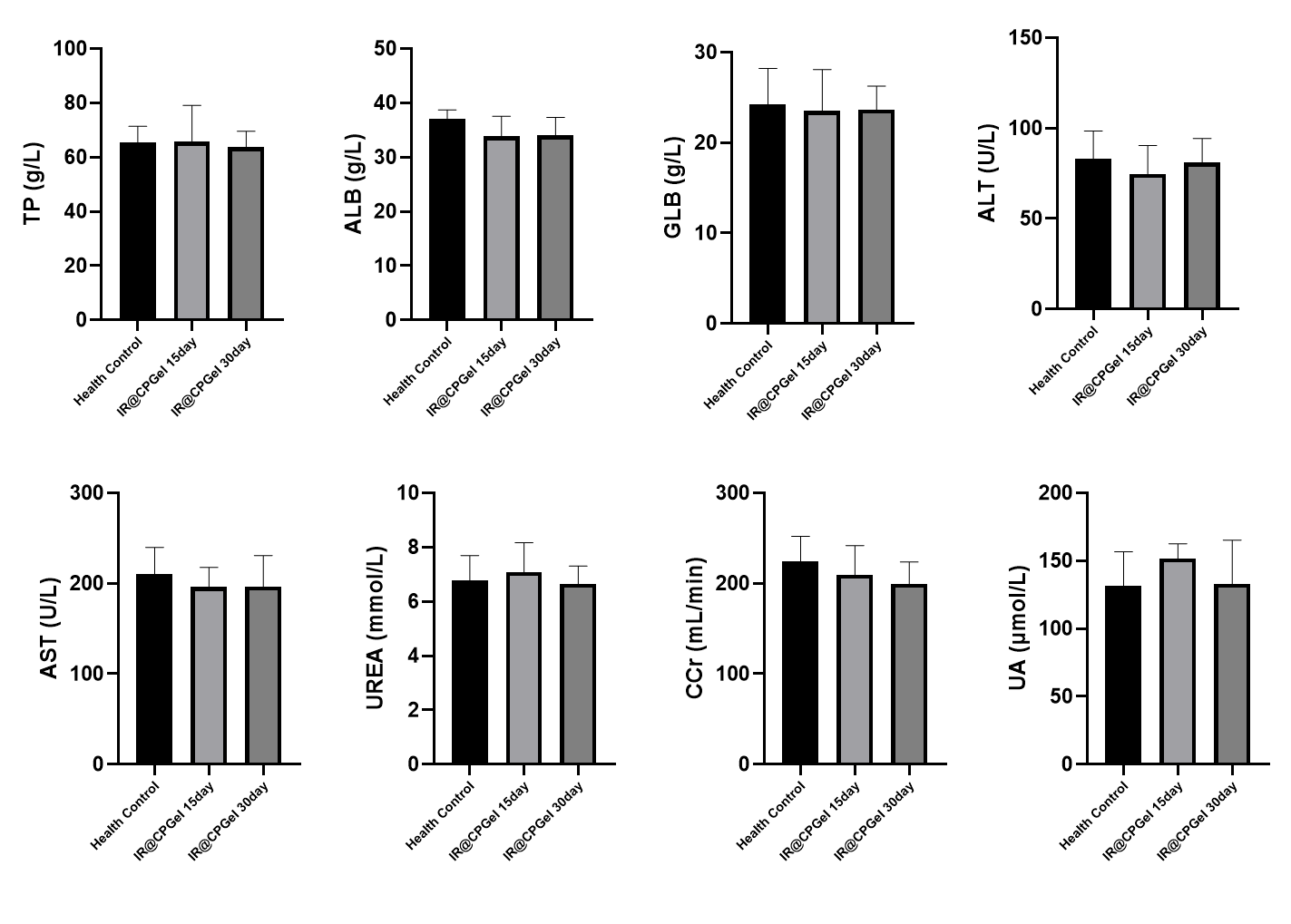


**FIGURE S6** Biochemical indices of mice major organs in 30 days after subcutaneous injection of IR@CPGel, All data are shown as mean ± S.D. (n = 3)
